# Outcomes of amoebic, fungal, and bacterial keratitis: A retrospective cohort study

**DOI:** 10.1371/journal.pone.0264021

**Published:** 2022-02-16

**Authors:** Caitlin A. Moe, Prajna Lalitha, N. Venkatesh Prajna, Jeena Mascarenhas, Muthiah Srinivasan, Manoranhan Das, Arun Panigrahi, Revathi Rajaraman, Gerami D. Seitzman, Catherine E. Oldenburg, Thomas M. Lietman, Jeremy D. Keenan

**Affiliations:** 1 Francis I. Proctor Foundation, University of California, San Francisco, California, United States of America; 2 Department of Ocular Microbiology, Aravind Eye Care System, Madurai, India; 3 Department of Cornea and External Diseases, Aravind Eye Care System, Madurai, India; 4 Department of Cornea and External Diseases, Aravind Eye Care System, Coimbatore, India; 5 Department of Ophthalmology, University of California, San Francisco, California, United States of America; 6 Department of Epidemiology & Biostatistics, University of California, San Francisco, California, United States of America; University of Toronto, CANADA

## Abstract

**Background:**

*Acanthamoeba* keratitis is challenging to treat and thought to result in poor outcomes, but very few comparative studies exist to assess whether ulcers caused by *Acanthamoeba* are worse than those caused by bacteria or fungus.

**Methods:**

In a retrospective cohort study, all cases of smear- or culture-proven *Acanthamoeba* keratitis diagnosed from January 2006 to June 2011 at an eye hospital in South India were identified from the microbiology database. Random samples of the same number of cases of bacterial and fungal keratitis, matched by year, were identified from the same database in order to compare outcomes between the three types of organism. The main outcomes were the time until the following events: re-epithelialization, discontinuation of antimicrobials, perforation/keratoplasty, elevated intraocular pressure, and new cataract.

**Results:**

The median time until re-epithelialization was 113 days for *Acanthamoeba* keratitis, 30 days for fungal keratitis, and 25 days for bacterial keratitis, and the median time until discontinuation of antimicrobial therapy was 100 days for *Acanthamoeba* keratitis, 49 days for fungal keratitis, and 40 days for bacterial keratitis. Compared to the other two organisms, *Acanthamoeba* ulcers took significantly longer to re-epithelialize (adjusted HR 0.4, 95% CI 0.3 to 0.6 relative to bacterial ulcers and HR 0.3, 95% CI 0.2 to 0.5 relative to fungal ulcers; overall p<0.001) and had significantly longer courses of antimicrobials (adjusted HR 0.3, 95% CI 0.2 to 0.6 relative to bacterial ulcers and HR 0.5, 95%CI 0.3 to 0.8 relative to fungal ulcers; overall p<0.001). No statistically significant difference was observed between the three organisms for the other time-to-event outcomes.

**Conclusions:**

Acanthamoeba keratitis was more difficult to treat and had worse clinical outcomes than bacterial or fungal ulcers, highlighting the lack of adequate treatment regimens for this infection.

## Introduction

*Acanthamoeba* keratitis is one of the most severe corneal infections [1]. *Acanthamoeba* cysts are extremely resistant to a variety of medications, resulting in long treatment courses [2]. Poor outcomes are common, likely a combination of inflammation directly related to the organism as well as medication toxicity [3, 4]. Many believe clinical outcomes of *Acanthamoeba* keratitis are worse than those of bacterial keratitis [5, 6]. While numerous case series have reported poor outcomes of *Acanthamoeba* keratitis, few studies have compared outcomes of different causative organisms of keratitis [7–9], and even fewer have compared visual outcomes between different organisms [10]. Here, we report the results of a retrospective cohort study from a single eye hospital in South India in which we compared clinical outcomes from *Acanthamoeba*, bacterial, and fungal keratitis. We hypothesized that *Acanthamoeba* keratitis would have worse clinical outcomes than the other two organisms.

## Methods

### Study design

In this retrospective cohort study, we identified all cases of smear- or culture-proven *Acanthamoeba* keratitis from the microbiology database at Aravind Eye Hospital in Madurai, India from January 2006 to June 2011 [11]. The Aravind Microbiology Laboratory keeps a database of the results of all smears and cultures performed for clinical care at the Madurai hospital location, regardless of result. As a general rule all patients presenting to Aravind Eye Hospital with a corneal ulcer (i.e., corneal stromal infiltrate, corneal epithelial defect, and ocular inflammation) are scraped for culture and smear. The same number of bacterial and fungal keratitis cases were randomly selected for each year of the study. No other matching (e.g., by age or sex) was performed when selecting random samples. Outcomes and risk factors were extracted from patients’ medical charts using a standardized data collection form. Data extractors were masked to the identity of the organism by having a separate chart reviewer obscure all references to microbiological diagnosis with adhesive paper. Patients were not excluded based on ocular comorbidities or visual status. A 6-month follow-up period was designated for all outcomes, counting time from the date of the first positive microbiologic test. The date of the first mention of the outcome in the medical record was recorded, with administrative censoring after 6 months. Clinical outcomes were coded as present if documented in the chart, and absent if not documented. For patients lost to follow-up before 6 months, clinical information from the time of last follow-up were analyzed with a censoring weight, as described below.

### Ethics

Ethical approval for this study was obtained from the Committee for Human Research at the University of California, San Francisco, and from the Institutional Review Board (IRB) at Aravind Eye Hospital, Madurai. The IRBs waived the requirement of informed consent for this retrospective study. Data were fully anonymized before analysis. This research adhered to the tenets of the Declaration of Helsinki.

### Clinical management

During the study period bacterial keratitis was typically treated with topical moxifloxacin monotherapy with or without topical corticosteroids, fungal keratitis with topical natamycin or voriconazole, and *Acanthamoeba* keratitis with polyhexamethylene biguanide (PHMB) monotherapy without topical corticosteroids. The details of antimicrobial therapy were not recorded for this study.

### Statistical analysis

Presenting visual acuity (i.e., patients wore their glasses if they had them) was recorded in the medical records in Snellen notation and transformed to logMAR acuity for all analyses; logMAR values of 1.9 and 2.3 were used to represent counting fingers and hand motion vision respectively, and values of 2.7 and 3.0 were extrapolated to represent vision of light perception and no light perception [12]. Kaplan-Meier survival curves and Cox-proportional hazards models were constructed for several time-to-event outcomes, including re-epithelialization, discontinuation of antimicrobials, perforation/therapeutic keratoplasty, intraocular pressure elevation ≥25mmHg, and new cataract (i.e., cataract not present at the time of initial diagnosis and documented at a subsequent visit). Missing data was dealt with by employing listwise deletion. Because of the likelihood that those participants lost to follow-up were different from those who remained in follow-up, we created inverse probability of censoring weights (i.e., the probability that the individual completed 6 months of follow-up) for the Cox-proportional hazards models, using the following baseline covariates as predictors of loss to follow-up: age, sex, residence in same state as eye hospital, payment method (self-pay vs. charity), history of eye trauma, history of past ocular surgery, history of diabetes, duration of symptoms, concurrent antibiotic use, concurrent corticosteroid use, concurrent antifungal use, presenting visual acuity, infiltrate depth extending to posterior third of cornea, presence of hypopyon, and presence of corneal perforation. The analysis sought to determine differences between the 3 organisms, and hence the significance test of interest was the p-value for 3-level organism variable from each model, with a significance level of 0.01 in a two-tailed test for each of the 5 survival analysis outcomes.

## Results

From January 2006 to June 2011, 115 *Acanthamoeba* keratitis cases were listed in the microbiological database at Aravind Eye Hospital-Madurai, of which 93 (81%) had medical records available for review. We randomly selected 115 bacterial and 115 fungal cases from the same time period and successfully located medical records for 95 (83%) of the bacterial cases and 103 (90%) of the fungal cases. The causative species have been reported previously; bacterial infections were predominantly *Streptococcus pneumoniae* (38%) and *Pseudomonas aeruginosa* (29%) and fungal infections were predominantly *Fusarium* (31%) and *Aspergillus* (25%) species [11]. Characteristics of patients and eyes at the time of the first positive microbiologic test are shown in Table 1. Concurrent participation in a separate clinical trial was noted for 16 (17%) patients with bacterial keratitis, 1 (1%) with fungal keratitis, and none with *Acanthamoeba* keratitis.

**Table 1:** Characteristics of included participants and eyes at the time of keratitis diagnosis, stratified by organism.

Characteristic	*Acanthamoeba* (N = 93)	Bacteria (N = 95)	Fungus (N = 103)	*P*-value^a^
Age, years	38 ± 16	50 ± 18	43 ± 16	<0.001
Female	40 (43%)	29 (31%)	41 (40%)	0.18
Symptom duration, days	31 ± 61	13 ± 39	10 ± 13	<0.001
Trauma	55 (59%)	60 (64%)	62 (60%)	0.86
Past ocular surgery	5 (5%)	22 (23%)	12 (12%)	0.001
Cataract extraction	4 (4%)	15 (16%)	9 (9%)	0.03
Keratoplasty	0 (0%)	2 (2%)	1 (1%)	0.53
Other^b^	1 (1%)	5 (5%)	2 (2%)	0.23
Contact lens wear	3 (3%)	2 (2%)	0 (0%)	0.17
Topical antibiotic use	58 (62%)	28 (30%)	48 (47%)	<0.001
Topical antifungal use	36 (39%)	19 (20%)	34 (33%)	0.02
Topical steroid use	8 (9%)	6 (6%)	6 (6%)	0.74
Infiltrate size, mm	5.6 ± 3.0	4.6 ± 3.3^c^	4.6 ± 3.1	0.007
Infiltrate depth posterior third	32 (34%)	42 (45%)	36 (35%)	0.29
Visual acuity, logMAR	1.73 ± 0.84	1.70 ± 0.97	1.40 ± 0.96	0.03

Values indicate mean ± standard deviation or number (percentage).

^a^ Kruskal-Wallis test for continuous variables and Fisher’s exact test for categorical variables.

^b^ Amniotic membrane (*Acanthamoeba* group, N = 1); dacryocystectomy (bacteria group, N = 1); retinal detachment repair (fungus group, N = 1); the remainder were unspecified.

^c^ Two bacterial ulcers missing baseline infiltrate size.

Loss to follow-up was considerable for this study population, with a median follow-up time of 1.9 months (IQR 0.5 to 6.7). Follow-up of at least 1 month was documented for 70 (75%) *Acanthamoeba* patients, 56 (59%) bacterial keratitis patients, and 62 (60%) fungal keratitis patients. There was not strong evidence of differential loss to follow-up by organism (Table 2). Nonetheless, visual acuity outcomes were not analyzed given the potential for bias due to the varying timing of the assessments.

**Table 2:** Baseline characteristics, stratified by follow-up status 1 month following diagnosis of infectious keratitis.

	*Acanthamoeba*		Bacteria		Fungus		
	Complete (N = 70)	LTFU (N = 23)	Complete (N = 56)	LTFU (N = 39)	Complete (N = 62)	LTFU (N = 41)	*P*-value
Age, years	37 ± 16	40 ± 16	50 ± 19	50 ± 18	45 ± 16	41 ± 17	0.32
Female	32 (46%)	8 (35%)	14 (25%)	15 (39%)	24 (39%)	17 (41%)	0.27
Visual acuity, logMAR	1.7 ± 0.9	1.8 ± 0.8	1.7 ± 0.9	1.8 ± 1.0	1.5 ± 1.0	1.2 ± 0.9	0.11
Infiltrate size, mm	5.7 ± 3.0	5.0 ± 3.0	4.4 ± 3.0	4.9 ± 3.6	5.3 ± 3.2	3.7 ± 2.7	0.06

Values indicate mean ± standard deviation or number (percentage). The P-value comes from the interaction of organism by follow-up status in a regression modeling each characteristic. LTFU = loss to follow-up.

**Fig 1:**
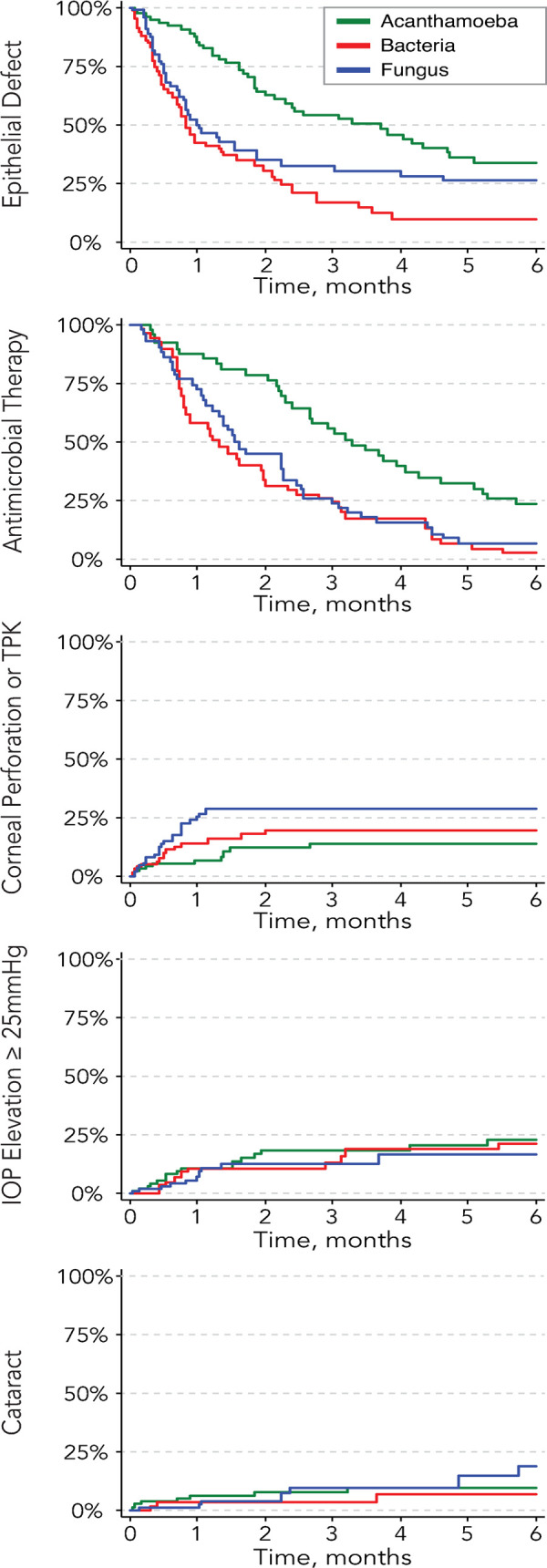
Kaplan-Meier survival curves of several outcomes, stratified by organism.

Kaplan-Meier curves for time until re-epithelialization, discontinuation of antimicrobials, perforation/therapeutic keratoplasty, cataract, and intraocular pressure elevation are shown in Fig 1. The results of the corresponding Cox proportional hazards models for each of these outcomes are shown in Table 3, adjusted for baseline covariates. Compared to the other two organisms, *Acanthamoeba* ulcers took significantly longer to re-epithelialize (adjusted HR 0.4, 95% CI 0.3 to 0.6 relative to bacterial ulcers and HR 0.3, 95% CI 0.2 to 0.5 relative to fungal ulcers; overall p<0.001) and had significantly longer courses of antimicrobials (adjusted HR 0.3, 95% CI 0.2 to 0.6 relative to bacterial ulcers and HR 0.5, 95%CI 0.3 to 0.8 relative to fungal ulcers; overall p<0.001). No statistically significant difference was observed between the three organisms for the other time-to-event outcomes.

**Table 3:** Association between organism and several time-to-event outcomes, after adjustment for baseline covariates.

Baseline characteristic	Re-epithelialization	Discontinuation of Antimicrobials	Corneal perforation/graft	Intraocular pressure ≥25mmHg	Cataract
**Organism**					
*Acanthamoeba*	Ref	Ref	Ref	Ref	Ref
Bacteria	3.5 (2.1 to 5.8)	3.0 (1.8 to 5.3)	1.5 (0.6 to 3.9)	1.2 (0.5 to 3.2)	0.4 (0.1 to 2.0)
Fungus	1.9 (1.2 to 3.2)	2.1 (1.3 to 3.4)	3.7 (1.6 to 9.0)	1.0 (0.4 to 2.8)	1.0 (0.3 to 4.1)
*P-value*	*P<0*.*001*	*P<0*.*001*	*P = 0*.*03*	*P = 0*.*74*	*P = 0*.*65*
**Baseline covariates**					
Age, per decade	0.8 (0.7 to 0.9)	1.0 (0.8 to 1.1)	1.1 (0.9 to 1.4)	1.1 (0.8 to 1.3)	1.2 (0.9 to 1.7)
Female sex	0.8 (0.6 to 1.2)	1.2 (0.8 to 1.7)	1.3 (0.7 to 2.4)	0.9 (0.4 to 2.0)	1.1 (0.4 to 2.9)
Vision, per line worse	1.0 (1.0 to 1.0)	1.0 (1.0 to 1.0)	1.1 (1.0 to 1.2)	1.0 (1.0 to 1.1)	1.0 (1.0 to 1.1)
Depth >66%	0.7 (0.4 to 1.0)	0.6 (0.4 to 0.9)	1.5 (0.7 to 3.2)	1.3 (0.6 to 2.9)	1.0 (0.2 to 5.2)
Infiltrate size, per mm	0.9 (0.8 to 1.0)	1.0 (0.9 to 1.1)	1.1 (1.0 to 1.2)	0.9 (0.8 to 1.1)	1.0 (0.8 to 1.4)
Symptom duration, wks	0.9 (0.9 to 1.0)	1.0 (0.9 to 1.0)	1.0 (1.0 to 1.2)	1.0 (1.0 to 1.1)	1.0 (1.0 to 1.1)
Antimicrobial use	0.6 (0.4 to 0.9)	0.8 (0.5 to 1.2)	1.0 (0.6 to 1.9)	1.7 (0.7 to 4.2)	0.4 (0.1 to 1.2)
Steroid use	1.0 (0.6 to 2.0)	1.0 (0.6 to 1.7)	0.7 (0.2 to 2.2)	1.0 (0.3 to 3.6)	<0.01 (<0.01-<0.01)

Values indicate hazard ratios and 95% confidence intervals except for the P-values, which provide results from a Wald test assessing a difference between the three organisms of interest. Statistical significance was defined as a P-value <0.001.

## Discussion

In this comparative observational study, eyes with *Acanthamoeba* keratitis re-epithelialized more slowly and remained on antimicrobial therapy for a longer duration of time than bacterial or fungal ulcers.

As depicted in Fig 1, re-epithelialization for cases of *Acanthamoeba* keratitis took a median of 113 days, which was about 4.5 times as long as bacterial ulcers (median 25 days) and 3.5 times as long as fungal ulcers (median 30 days). Perhaps related to this, *Acanthamoeba* cases were continued on antimicrobial therapy for a median of 100 days, which was 2.5 times as long as bacterial ulcers (median 40 days) and approximately twice as long as fungal ulcers (median 49 days). These findings are consistent with prior studies of *Acanthamoeba* keratitis where months-long treatment courses are the norm [13].

Although the association did not reach statistical significance, perforation and/or therapeutic keratoplasty was more common in fungal than bacterial or *Acanthamoeba* ulcers. This finding is consistent with previous studies have reported high rates of corneal perforation and/or need for therapeutic keratoplasty in fungal keratitis [14–16]. Others have found that the rate of corneal perforation or need for keratoplasty in *Acanthamoeba* keratitis may depend on prior exposure to corticosteroids. In one study, 43% of *Acanthamoeba* keratitis cases with prior corticosteroid use required therapeutic keratoplasty while only 9% without prior corticosteroids needed keratoplasty [13]. The importance of topical corticosteroids was not confirmed in the present study, although relatively few patients were exposed to this risk factor.

This study should be interpreted in light of several limitations. A chief limitation is the retrospective nature of the study and inherent missing data, loss to follow-up, and potential for misclassification error, all of which can result in bias. A relatively large number of patients were lost to follow-up before 6 months, making comparisons of post-treatment visual acuity unreliable. Such high loss-to-follow-up rates are not entirely surprising since the average duration of therapy is 1–2 months and there was no incentive offered to participants to return. We attempted to address the major potential confounders in a multivariable analysis, but unmeasured confounders (e.g., the exact species of each of the 3 major organisms, or ocular comorbidities such as limbal stem cell deficiency, thyroid eye disease, or exposure keratopathy) could have resulted in bias. We addressed the loss to follow-up by applying a censoring weight to account for the possibility of differential loss to follow-up between the three types of ulcers. Outcomes such as a newly developed cataract or glaucoma may have been missed or gone undocumented in the chart because the data was not collected prospectively. The details of treatment were not recorded specifically for each patient. However, because the data come from the cornea clinic of a single eye hospital, all patients with a particular organism were treated according to similar protocols. Moreover, in our causal framework the treatment is intimately tied to the organism, and if anything, might be a mediating factor rather than a confounder. *Acanthamoeba* was typically treated with biguanide monotherapy. While monotherapy is commonly used by some cornea specialists, others prefer a combination of agents [17]. It is unclear whether the use of combination therapy would have changed the results. Furthermore, the study was conducted in a single eye hospital in South India where contact lens is infrequent. These findings may not be generalizable to contact lens wearers who develop a corneal ulcer, to milder ulcers that may not have presented to a tertiary eye hospital, to culture- or smear-negative ulcers, or to settings outside of South India.

## Conclusions

Clinical experience suggests that *Acanthamoeba* keratitis results in worse outcomes than fungal or bacterial keratitis. In this retrospective cohort study, we confirmed that, on average, patients with *Acanthamoeba* ulcers had longer-re-epithelialization times and longer durations of antimicrobial therapy. Prospective interventional trials are a challenging prospect for *Acanthamoeba* keratitis given the paucity of cases, but would be helpful to explore optimal treatment strategies.

## Supporting information

S1 Data Deidentified data. (CSV)
